# Modulation of gut microbiota by Gardeniae Fructus oil exerts TLR4/NF-κB/NLRP3 pathway-mediated antidepressant effects based on transcriptomics and fecal transplantation

**DOI:** 10.3389/fphar.2025.1635897

**Published:** 2025-08-01

**Authors:** Man Han, Yujing Zhou, Xinyu Gao, Xixi Cheng, Ling Deng, Shilin Ji, Zhengheng Li, Yu Cai, Chunchao Yan, Yunzhong Chen

**Affiliations:** ^1^Faculty of Pharmacy, Hubei University of Chinese Medicine, Wuhan, China; ^2^Hubei Provincial Research Center for TCM Health Food Engineering, Hubei University of Chinese Medicine, Wuhan, China; ^3^Hubei Shizhen Laboratory, Hubei University of Chinese Medicine, Wuhan, China

**Keywords:** Gardeniae Fructus oil, depression, chronic unpredictable mild stress, fecal microbiota transplantation, transcriptomics, gut-brain axis, TLR4/NF-κB/NLRP3

## Abstract

**Background:**

Although our team has demonstrated the antidepressant effect of Gardeniae Fructus oil (OGF) in the early stages, the mechanism of whether OGF works by regulating the gut microbiota is not clear. This study aims to elucidate OGF’s gut-brain axis mechanism in depression.

**Methods:**

Chronic unpredictable mild stress (CUMS) was used to establish a depressed mouse model, and the depression-like behavior of mice was observed by behavioral tests after antibiotic pretreatment and fecal microbiota transplantation (FMT). HE staining was used to observe the pathological changes in the hippocampus and colon; ELISA was used to detect the content of neurotransmitters and pro-inflammatory factors; Western blot was used to detect the expression of colonic tight junction proteins. The signaling pathways regulating the antidepressant properties of OGF were obtained by transcriptome sequencing analysis and validated at the protein level.

**Results:**

Compared with the CON group, mice in the CUMS group showed significant depressive-like behavior, pathological damage to the hippocampus and colon tissues, significant decrease in levels of 5-HT, DA, and BDNF in the hippocampus, significant increase in levels of IL-1β, IL-6, TNF-α, DAO, and LPS in serum, significant decrease in colonic tight junction protein expression, and significant increase in protein expression of TLR4, p-NF-κB, NLRP3, ASC, and IL-1β in the hippocampus (*P* < 0.01); Compared with the CUMS group, the FMT group could effectively improve the above situation (*P* < 0.05, *P* < 0.01), whose therapeutic effect was second only to the OGF group (*P* < 0.01), while ABX + OGF group did not show obvious therapeutic effect.

**Conclusion:**

OGF might exert antidepressant effects by modulating gut microbiota and mediating the hippocampal TLR4/NF-κB/NLRP3 pathway.

## Introduction

Depression, a psychiatric disorder characterized by significant and persistent low mood, is projected to become the foremost global burden of disease by the year 2030 (Monroe and Harkness, 2022). In 2020, the prevalence of depression and anxiety increased by 25% due to the impact of the COVID-19 pandemic (Herrman et al., 2022). At present, the prevailing clinical approach to depression involves the administration of medications such as fluoxetine, paroxetine, and venlafaxine. However, these antidepressants have been shown to have significant side effects and long-term use affects liver and kidney function (Cipriani et al., 2018). This makes the search for more effective and safer alternatives from botanic resources imminent. Studies have reported that oils extracted from natural plants can alleviate depression-like symptoms in several ways (Zhang et al., 2021).

Gardeniae Fructus, a traditional Chinese medicinal and food plant, is utilized both as a natural additive in the food industry and as a core Chinese herb in numerous antidepressant herbal formulations (Liu et al., 2023). Research has demonstrated that OGF contains a significant number of antidepressant components, including linoleic acid, α-linolenic acid, squalene, β-sitosterol, and saffron acid (Ruan et al., 2019). However, the full potential of OGF in the treatment of depression remains to be fully realized.

In previous studies, our team has demonstrated that OGF can ameliorate depression-like behavior in chronic unpredictable mild stress (CUMS) mice, and 16S rDNA high-throughput sequencing results have shown that OGF can improve intestinal microbiota dysbiosis in depressed mice (Han et al., 2024). Nowadays, a large number of studies have demonstrated the involvement of the microbiota-gut-brain (MGB) axis in the process of depression development, and that fecal microbiota transplantation (FMT) and supplementation with probiotic preparations can improve depressive symptoms in patients or animals (Socała et al., 2021). However, it is not clear whether OGF exerts its antidepressant effects by regulating the intestinal microbiota. The mechanism of action is unknown.

The current study sought to address two key points, first, whether OGF exerts its antidepressant effects with the help of intestinal flora, and second, to identify the signaling pathways that were modulated in the treatment. Therefore, the present study continues to use transcriptomics and bioinformatics to determine the relevant signaling pathway of the antidepressant effect of OGF and deeply verifies the antidepressant mechanism of OGF based on the gut-brain axis through FMT, with a view to providing a scientific basis for further research and development.

## Materials and methods

### Materials

Gardeniae Fructus (Batch No.: 20220101) was obtained from Hubei Qiangkang Traditional Chinese Medicine Slices Co., Ltd. (Ezhou, China). OGF was obtained by petroleum ether extraction of Gardeniae Fructus for three times. The ELISA kits with 5-HT (MU30036), DA (MU30456), BDNF (MU30097), IL-1β(MU30369), IL-6 (MU30044), TNF-α (MU30030), DAO (MU30134), LPS (MU30451) were purchased from Bioswamp. Primary antibodies against β-actin (GB15003), occludin (GB111401), TLR4 (GB11519), NLRP3 (GB114320), ASC (GB113966), and IL-1β (GB11113) as well as the secondary antibody (GB23303) were obtained from Servicebio Technology Co., Ltd. (Wuhan, China). Primary antibodies against zonula occludens-1 (ZO-1; AF5145) and cleaved caspase-1 (AF4005) were obtained from Affinity Biosciences (Jiangsu, China). Primary antibodies against claudin-1 (28674-1-AP) and NF-κB (80979-1-RR) were obtained from Proteintech Biotechnology Co., Ltd. (Wuhan, China). The primary antibody against p-NF-κB (WL02169) was obtained from Wanleibio Co., Ltd. (Shenyang, China).

### Animals and experimental design

Sixty-three male ICR mice (4 weeks old, 21–23 g) were obtained from Hunan SJA Laboratory Animal Co., Ltd. (Changsha, China) under license number SCXK (Xiang) 2019-0004. All animal procedures were approved by the Animal Ethics Committee (Approval No. HLK-20240307-001). During the entire study, all mice were housed under an ambient temperature of 22°C ± 2°C, relative humidity of 55% ± 5%, and a 12-h light/dark cycle. Mice received a standard diet and water *ad libitum*.

Mice were kept in individual cages and exposed to the CUMS program (Willner, 2017; Zhou et al., 2022). Each mouse randomly received one long-term and one short-term stressor, and the same stressors were not used for two consecutive days. This protocol included the following stressors: eight long-term stressors (for 24 h) such as fasting, water deprivation, white noise (90 dB), stroboscopic light source, odor stimulation, 45° cage tilting, empty cage, and wet bedding; six short-term stressors such as restraint (6 h), swimming in cold water (4°C for 5 min), heat stress in an oven (55°C for 10 min), cage shaking (2 h), tail pinching (2 min), and electrical stimulation (0.6 mA, 50 s).

**FIGURE 1:**
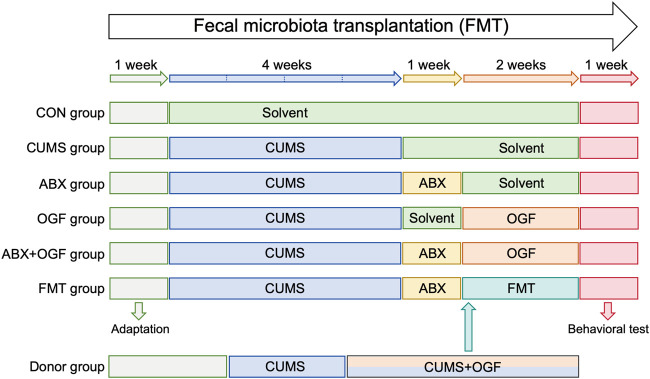
Experimental design of FMT.

After acclimatization, mice were randomly divided into 6 groups (n = 9): control + solvent group (CON group), CUMS + solvent group (CUMS group), CUMS + antibiotic group (ABX group), CUMS + OGF 122 mg/kg group (OGF group), CUMS + antibiotic + OGF 122 mg/kg group (ABX + OGF group), CUMS + antibiotic + fecal bacteria transplantation group (FMT group). The recommended clinical dosage of Gardeniae Fructus was 10 g/60 kg per day, and the dose of OGF (122 mg/kg) was calculated through the body surface area conversion formula and the oil extraction rate. The determination of this dosage had been screened through previous pharmacological studies. Except for the CON group, the mice in the other five groups were housed in single cages. After the 4-week CUMS procedure, mice in the ABX, ABX + OGF, and FMT groups received 1-week antibiotic pretreatment. The body weight of each mouse was monitored regularly, and finally behavioral tests were performed. The specific experimental flow is shown in Figure 1.

Source of fecal bacteria: The donor group (n = 9) received a 6-week CUMS procedure, which began with gavage of 122 mg/kg OGF in the third week. After 2 weeks of OGF treatment, fresh feces were collected daily from the mice in the Donor group and immediately made into a fecal bacterial suspension. The fresh feces were added to pre-cooled PBS (100 mg feces/1 mL of buffer) and vortexed for 2 min, and the supernatant was collected after centrifugation at 800 *g* for 5 min. Each mouse in the FMT group received 100 μL of fecal bacterial suspension (Yao et al., 2023).

### Animals and experimental design

A 7-day antibiotic pretreatment was developed according to previously described methods (Olson et al., 2018). Mice were given antibiotic cocktails daily consisting of vancomycin hydrochloride (50 mg/kg), neomycin sulfate (100 mg/kg), and metronidazole (100 mg/kg). Amphotericin-B (1 mg/kg) was added to the cocktail during the first 3 days. Ampicillin sodium (1 mg/mL) was provided in drinking water.

### Behavioral tests

#### Sucrose preference test

The sucrose preference test (SPT) is commonly used to assess the degree of anhedonia by observing sucrose consumption, which is considered as a core symptom of depression. The SPT was performed at 2-week intervals throughout the experiment, encompassing the adaptation period (week 0), the pre-treatment phase (week 2), the treatment phase (week 4), and the post-treatment phase (week 6).

Firstly, all mice were acclimatised to one bottle of 1% sucrose water and one bottle of pure water for a 48-h period. Thereafter, the position of both bottles was exchanged every 12 h. Then, mice were deprived of water for 24 h. Subsequently, each mouse was given a bottle with 60 mL pure water and a bottle with 60 mL 1% sucrose solution for 24 h, where the positions of both bottles were also switched after 12 h. The consumption of sucrose solution and pure water was measured by weighing the bottles before and after the experiment. Sucrose preference was calculated according to the following formula: sucrose preference (%) = sucrose intake (g)/[water intake (g) + sucrose intake (g)] × 100%.

#### Novelty-suppressed feeding test

The novelty-suppressed feeding test (NSFT) is widely used to evaluate behavioral indicators of depression and anxiety. The test was conducted over a period of 2 days: on the first day, mice were individually placed in white open boxes for a duration of 10 min in order to acclimatize and were then fasted for a period of 24 h. On the second day, 2–3 food pills were placed in the center of the open box, and then, mice were slowly placed in the corner of the box. The test lasted for 5 min and the feeding latency period was recorded, which was the time from the start of the test until the mice could be heard gnawing. After each test, the open box was meticulously sanitized with 75% ethanol in order to preclude residual odors from compromising the integrity of the subsequent test.

#### Forced swimming test

The forced swimming test (FST) was developed to assess the degree of behavioral desperation of animals in distress. Mice were placed alone into a glass cylinder (height: 25 cm, diameter: 12 cm) containing water (25°C ± 1°C, at a depth of 15 cm) and were forced to swim for 6 min. The total immobility time in the last 5 min was analyzed by SuperFst 3.0 (NJKEWBIO, Nanjing, China). Mice were deemed to be immobile if they floated in water without displaying any signs of struggling, or if they moved only slightly to keep their heads above the surface. Between tests, glass cylinders were cleaned, and the water inside was replaced.

#### Tail suspension test

The tail suspension test (TST) was developed to assess the degree of desperation of animals under adversity. Mice were secured with tape 1 cm from the tip of the tail and kept suspended from the test apparatus for 6 min. After 1 min of acclimatization, the immobility time of each mouse was measured during the last 5 min by SuperTst 3.0 (NJKEWBIO). Mice were considered immobile when they hung passively without struggling.

#### Open field test

The open field test (OFT) was used to evaluate the autonomous motor ability, exploratory behavior, and anxiety state of animals. Mice were gently placed in the center of an open field box (50 × 50 × 40 cm) and allowed to explore the inside freely for 5 min. At the same time, the activity behavior was tracked by a video camera and further analyzed by KEMaze (NJKEWBIO) to obtain relevant parameters, including the total distance traveled and the time spent in the center area. The central area was thus defined as the four squares situated in the medial plane of the box. The bottom half of the box was thus subdivided into sixteen equally large squares.

#### Elevated plus-maze test

The elevated plus-maze test (EPM) was used to examine the anxiety state of mice, using an apparatus consisting of a central platform and four mutually perpendicular arms (5 × 30 cm). Two opposite arms were surrounded on three sides by 20-cm-high walls (closed arm), while the other two opposite arms were surrounded by no obstacles (open arm). A mouse was gently placed in the center of this maze in an orientation facing the open arm and was allowed to freely access all four arms for 5 min. Open-arm time ([open arm time]/[total arm time] × 100%) was assessed.

### Sample collection

After behavioral tests, mice were fasted for 12 h. After anesthetizing mice with 5% isoflurane (KW-MZJ-1, NJKEWBIO), their blood was collected from the orbital venous plexus and centrifuged (4°C, 1000 × *g*, 15 min), and serum was stored at −80°C for later testing. Subsequently, hippocampus and colon were collected, frozen in liquid nitrogen, and stored at −80°C. In addition, portions of brain and colon were collected and fixed in 4% paraformaldehyde.

### Histopathological examination

Brain and colon tissues of mice were fixed in 4% paraformaldehyde solution. Subsequently, tissues were embedded in paraffin wax and sectioned for hematoxylin-eosin (HE) staining. The CA1 and CA3 regions of hippocampus and colon were visualized using a microscope, and the depth of the colonic crypt was measured.

### Enzyme-linked immunosorbent assay

The hippocampal tissue was mixed with pre-cooled PBS for homogenization, centrifuged at 10,000 × *g* at 4°C for 10 min, and the supernatant was collected. Enzyme-linked immunosorbent assays (ELISA) were performed according to the instructions provided by the ELISA kit. 5-hydroxytryptamine (5-HT), dopamine (DA), brain-derived neurotrophic factor (BDNF) in hippocampus and interleukin-1β (IL-1β), interleukin-6 (IL-6), tumor necrosis factor-α (TNF-α), diamine oxidase (DAO), lipopolysaccharide (LPS) in serum were detected according to the instructions of the kit. Briefly, samples and standards were added to pre-coated plates, followed by incubation with specific detection antibodies. After washing, a substrate solution was added, and the reaction was stopped upon color development. Absorbance was measured at 450 nm by using the microplate reader.

### Identification of signaling pathways based on preliminary experiments

#### Transcriptome sequencing of hippocampus

In the pre-experiment about the pharmacology of OGF, the hippocampus of 3 mice were randomly selected as samples for sequencing analysis from the CON_0_ group, CUMS_0_ group and OGF-M_0_ group (OGF medium-dose group, 122 mg/kg), respectively. The pre-experiment process is shown in Supplementary Figure S1. The sequencing process sequentially included: total RNA extraction, total RNA quality testing, mRNA enrichment, mRNA fragmentation, double-stranded cDNA synthesis, end repair, A-addition, splice addition, fragment selection and PCR amplification of library fragments, library QC, and on-board sequencing on Illumina platform.

#### Differential expression gene analysis

Gene expression level was measured based on FPKM and gene differential expression analysis was performed using DESeq (1.10.1). P-values were corrected by Benjamini and Hochberg method to obtain FDR values (i.e., padj). Using |FoldChange| ≥ 1.5 and padj <0.05 as the screening condition, the differentially expressed genes (DEGs) were obtained between the CUMS_0_ group vs. the CON_0_ group, and the OGF_0_ group vs. the CUMS_0_ group. The upregulated and downregulated DEGs of the two groups were crossed with each other and intersections were taken to finally obtain the DEGs, which were regulated by OGF in the treatment of CUMS depressed mice.

#### Screening of depression-related targets

Depression-related disease targets were obtained by searching “Major Depressive Disorder” in three databases, including Genecards (https://www.genecards.org/), OMIM (https://omim.org/) and CTD (https://ctdbase.org/). In the Genecards and OMIM databases, a correlation score >4 times the median was used as a screening criterion. Ultimately, disease targets from the three databases were combined and duplicate targets were removed.

#### Screening of DEGs in genechips

The GEO database (https://www.ncbi.nlm.nih.gov/geo/) was searched for “Major Depressive Disorder”, the species was set to “*Homo sapiens*,” and the samples were from brain tissues. The GSE44593 dataset was obtained after screening. Four pairs of samples from depressed patients and controls were selected for gene chip quality analysis and screening of DEGs by the GEO2R analysis, and the screening conditions were the same as above.

#### qPCR detection of gene expression

Total RNA of hippocampus was extracted by Trizol method. After reverse transcription, fluorescent quantitative PCR was performed. β-actin was used as reference gene, and the relative expression of target gene was calculated by 2^−ΔΔCT^ method. Primer synthesis information is shown in Table 1.

**TABLE 1:** Sequence of PCR-related primers for each gene.

Gene name	Primer Sequence (5′-3′)	Length (bp)
TLR4	TGA​GGA​CTG​GGT​GAG​AAA​TGA​GC	223
	CTG​CCA​TGT​TTG​AGC​AAT​CTC​AT	
NF-κB p65	GCA​GAA​AGA​AGA​CAT​TGA​GGT​GTA​T	229
	GCG​ATC​ATC​TGT​GTC​TGG​CA	
NLRP3	ATG​ACT​TTC​CAG​GAG​TTC​TTC​GC	185
	CCA​AAG​AGG​AAT​CGG​ACA​ACA​A	
IL-1β	AGG​CTC​CGA​GAT​GAA​CAA​CAA​A	206
	GTG​CCG​TCT​TTC​ATT​ACA​CAG​GA	
β-actin	GTG​ACG​TTG​ACA​TCC​GTA​AAG​A	287
	GTA​ACA​GTC​CGC​CTA​GAA​GCA​C	

### Western blotting

Total proteins from colon and hippocampal tissues were extracted separately, and the protein concentration of each group was determined by BCA kit and adjusted to the same value. Add the protein up-sampling buffer and boil to obtain the up-sampled samples. The samples were separated by SDS-PAGE electrophoresis and membrane transfer. The membranes were blocked with 5% skimmed milk for 2 h, washed 6 times with TBST for 5 min each, and incubated with primary antibody at 4°C overnight. The next day, the membrane was washed and incubated in goat anti-rabbit secondary antibody (1:10,000) for 1 h at room temperature, then washed again. After ECL chemiluminescence detection, the optical densities were analyzed by ImageJ software and normalized to β-actin to quantify other protein levels.

Among them, primary antibodies for colon tissue included ZO-1 (1:3 000), occludin (1:5 000), claudin-1 (1:5 000), β-actin (1:10,000), and for hippocampal tissue TLR4 (1: 5 000), NF-κB (1:3 000), p-NF-κB (1:1 500), NLRP3 (1:3 000), ASC (1:5 000), cleaved caspase-1 (1:1 000), IL-1β (1:5 000), and β-actin (1:10,000).

### Statistical analysis

All data were analyzed using SPSS 26.0 (IBM, Chicago, IL, United States) and are expressed as mean ± SD. Comparisons between groups were analyzed by one-way analysis of variance (ANOVA), with *LSD* method for significant differences, and *Tambane’s T2* method for test of significant differences. *P* < 0.05 was considered to indicate statistically significant differences.

## Results

### Effect of FMT on body weight and depressive-like behavior in mice

**FIGURE 2:**
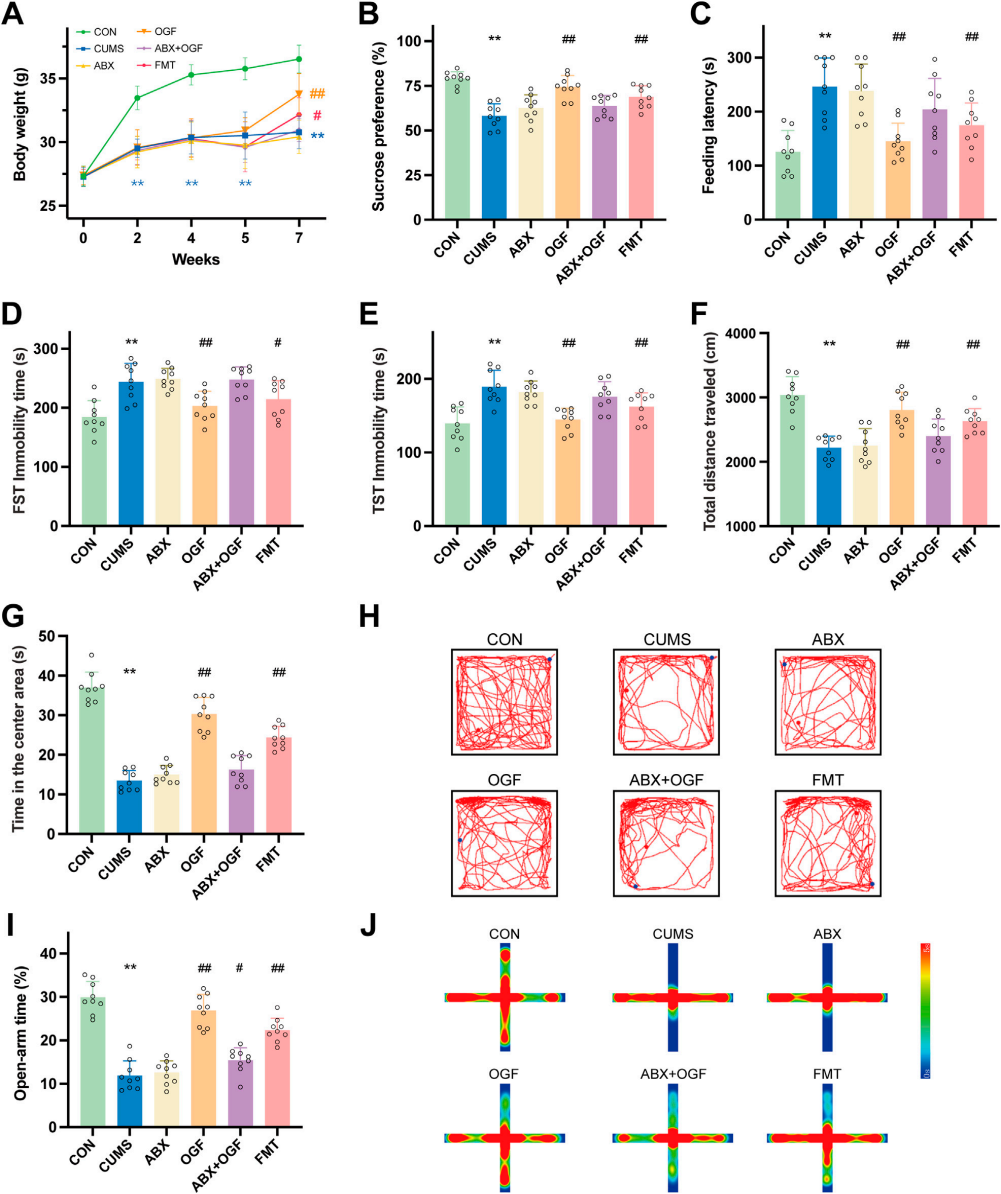
Effects of FMT on depression-like behavior in chronic unpredictable mild stress (CUMS)-induced mice. **(A)** Changes of body weight. **(B)** Sucrose preference rate at the end of the experiment. **(C)** Feeding latency in novelty-suppressed feeding test (NSFT). **(D)** Immobility time in forced swimming test (FST). **(E)** Immobility time in tail suspension test (TST). **(F)** Total distance traveled in open field test (OFT). **(G)** Time spent in the center area in OFT. **(H)** Motion trajectories in OFT. **(I)** Open-arm time in the elevated plus-maze (EPM). **(J)** Heat map of the motion trajectory in EPM, where the vertical axis represents the open arm and the horizontal axis represents the closed arm. Values are expressed as means ± SD (n = 9). ^*^ *P* < 0.05, ^**^ *P* < 0.01 *versus* CON group; ^#^ *P* < 0.05, ^##^ *P* < 0.01 *versus* CUMS group. The symbols of significance in the line graph correspond to each group by color.

During the first 4 weeks of CUMS modeling (Figure 2A), the body weights of the other five groups of mice were significantly decreased compared to the CON group (*P* < 0.01). At the 5th week, there was a slight decrease in body weight in ABX, ABX + OGF, and FMT mice after antibiotic treatment. After 2 weeks of treatment, the body weight of mice in the OGF and FMT groups increased significantly compared to the CUMS group (*P* < 0.05, *P* < 0.01), and the mice in the OGF group recovered their body weight better than those in the FMT group; however, the body weights of mice in the ABX and ABX + OGF groups were not significantly different from those in the CUMS group (*P* > 0.05).

Before the end of the experiment, behavioral tests such as SPT, NSFT, FST, TST, OFT, and EPM were administered (Figures 2B–J). Mice in the CUMS group showed significant depression-like behavior compared with the CON group (*P* < 0.01); the depression-like behavior of mice in the OGF group and the FMT group improved significantly after OGF treatment or transplantation of fecal bacteria from mice that had received OGF treatment compared with the CUMS group (*P* < 0.05 or *P* < 0.01), and the improvement in the OGF group was better than that in the FMT group. Compared with the CUMS group, the ABX and ABX + OGF groups of mice after antibiotic treatment or antibiotic treatment followed by OGF treatment mostly did not show significant improvement in depression-like behaviors.

### Effect of FMT on hippocampal tissue damage in CUMS-induced mice

**FIGURE 3:**
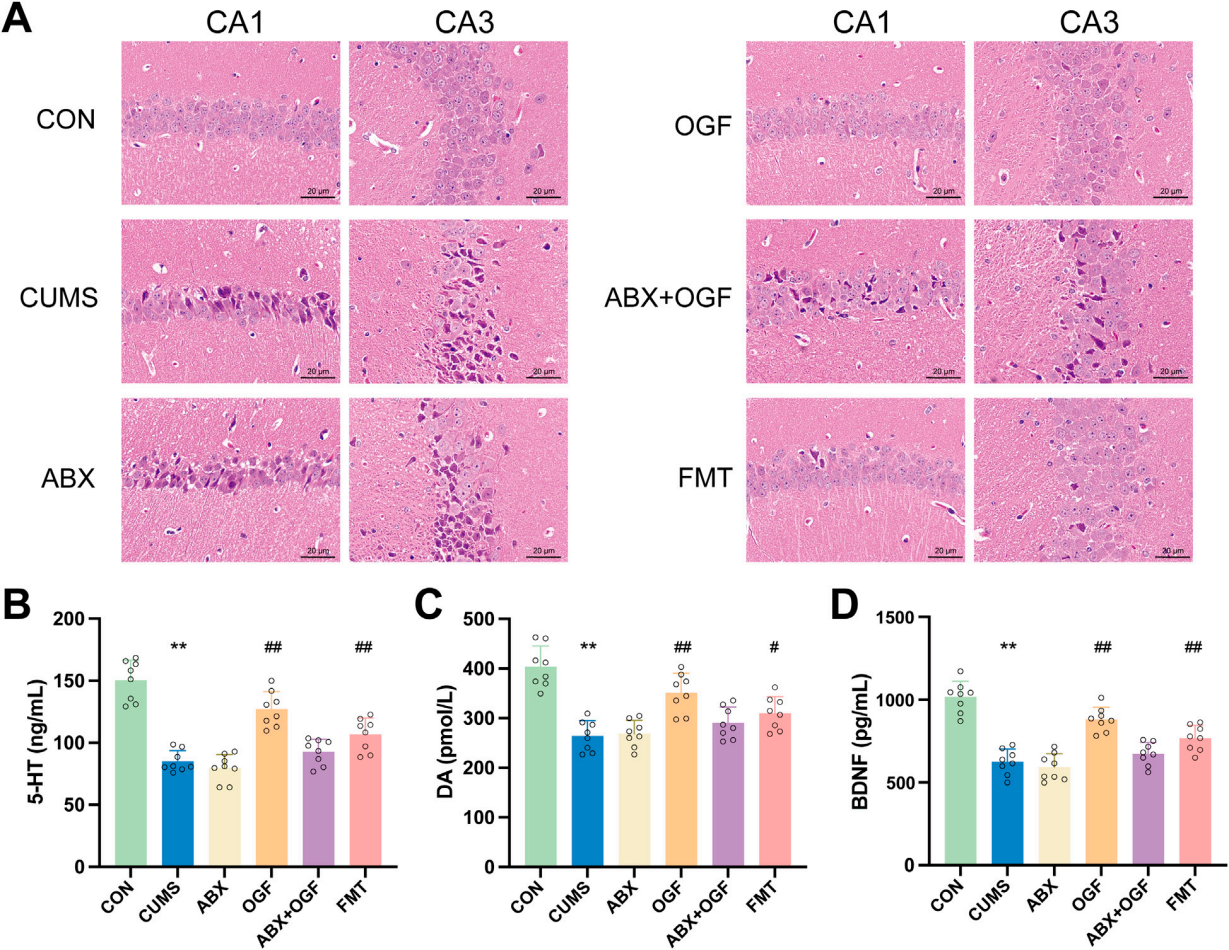
Effects of FMT on hippocampal damage and the levels of 5-HT, DA and BDNF in CUMS-induced mice. **(A)** Hematoxylin-eosin (HE) stained slices to observe the damage of CA1 and CA3 regions in the hippocampus (×200). **(B)** 5-HT **(C)** DA **(D)** BDNF. Values are expressed as means ± SD (n = 9). ^*^ *P* < 0.05, ^**^ *P* < 0.01 *versus* CON group; ^#^ *P* < 0.05, ^##^ *P* < 0.01 *versus* CUMS group.

As shown in Figure 3A, the neuronal cells in the CA1 and CA3 regions in the hippocampus of mice in the CON group were regularly arranged, normal in morphology and structure, with no apparent pathology. Compared with the CON group, the number of neurons in the CA1 and CA3 areas of the hippocampus in the CUMS group was reduced and disorganized, and some of the cells had abnormal morphology and structure, blurred outlines, and the nuclei of the cells were deeply stained and severely shrunken. Compared with the CUMS group, the pathological damage to the hippocampal tissue was significantly improved in the OGF and FMT groups, and the degree of pathological damage to the hippocampal tissue was partially attenuated in the ABX + OGF group, while no significant improvement was observed in the ABX group.

### Effects of fecal microbiota transplants on BDNF in the hippocampus in CUMS-induced mice

As shown in Figures 3B–D, the levels of 5-HT, DA, and BDNF in the hippocampus of mice in the CUMS group were significantly reduced compared with those in the CON group (*P* < 0.01). Compared with the CUMS group, the levels of 5-HT, DA, and BDNF in the hippocampus of mice in the OGF and FMT groups were significantly restored (*P* < 0.05, *P* < 0.01), with the restoration effect in the OGF group being superior to that in the FMT group. In addition, there were no significant differences in 5-HT, DA, and BDNF levels between the ABX group and the CUMS group. Although there was a slight reversal trend in the ABX+OGF group, it was not statistically significant.

### Effect of fecal microbial transplantation on colonic barrier permeability in CUMS-induced mice

#### Effect of fecal microbial transplantation on damaged colon tissue in CUMS-induced mice

**FIGURE 4:**
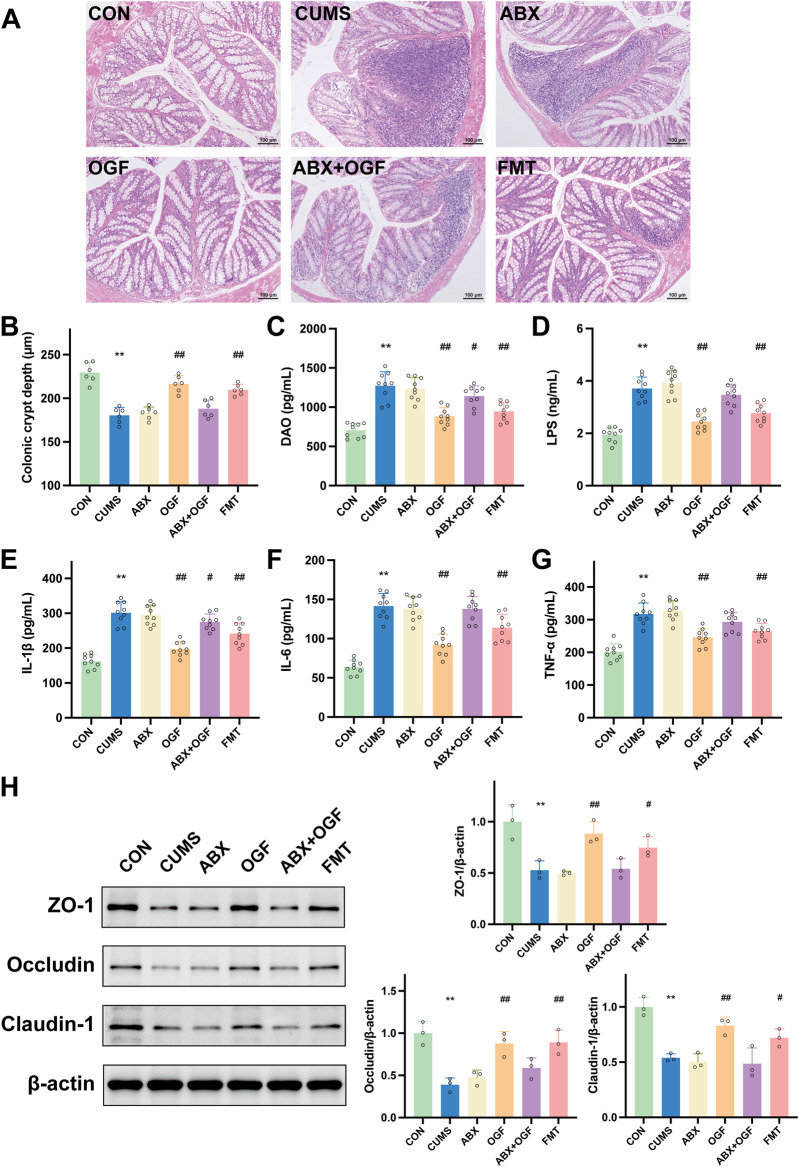
Effects of FMT on the permeability of the intestinal barrier in CUMS-induced mice. **(A)** HE stained slices showing histopathological changes in the colon (×100). **(B)** Crypt depth of the colon. **(C)** The diamine oxidase (DAO) level in serum. **(D)** The lipopolysaccharide (LPS) level in serum. **(E)** IL-1β. **(F)** IL-6. **(G)** TNF-α. **(H)** Effects of FMT on the expression of tight junction proteins, including ZO-1, occludin, and claudin-1. ^*^ *P* < 0.05, ^**^ *P* < 0.01 *versus* CON group; ^#^ *P* < 0.05, ^##^ *P* < 0.01 *versus* CUMS group.

In Figure 4A, the morphology and structure of the colon in the CON group was intact and normal, while the CUMS group had inflammatory cell infiltration in the mucosal layer and the colonic crypts were significantly disrupted compared with the CON group. Compared with the CUMS group, the colonic tissue structure of the mice in the OGF group and the FMT group was significantly improved and the infiltration of inflammatory cells was significantly reduced, while the pathology in the ABX group and the ABX + OGF group was not significantly improved. The same result was also reflected in the depth of the crypts in the colon of all the groups of mice (Figure 4B).

#### Effects of fecal microbial transplantation on serum DAO, LPS, IL-1β, IL-6, and TNF-α in CUMS-induced mice

DAO and LPS are biochemical indicators of gut barrier function, and IL-1β, IL-6 and TNF-α are major pro-inflammatory cytokines (Mohamad Nor et al., 2021). In Figures 4C–G, the serum levels of DAO, LPS, IL-1β, IL-6, and TNF-α in mice in the CUMS group were significantly increased compared with those in the CON group (*P* < 0.01); the levels of DAO, LPS, IL-1β, IL-6, and TNF-α in mice in the OGF group and the FMT group were significantly decreased compared with those in the CUMS group (*P* < 0. 05, *P* < 0.01), and the levels in the OGF group had a greater tendency to regress than those in the FMT group; compared with the CUMS group, there was no significant difference in the levels of each index in the serum of mice in the ABX group (*P* > 0.05), and there was a weak tendency to regress in the ABX + OGF group.

#### Effect of fecal microbial transplantation on ZO-1, occludin and claudin-1 proteins in mouse colon tissue

In the intestinal mucosal barrier, the tightly interconnected intestinal epithelial cells are the most important part of the mechanical barrier, with ZO-1, occludin, and claudin-1 as the key tight junction proteins (Wu et al., 2022). As shown in Figure 4H, the protein expression levels of ZO-1, occludin, and claudin-1 were significantly lower in the colon tissue of the CUMS group compared with the CON group (*P* < 0. 01); the protein expression levels of ZO-1, occludin, and claudin-1 were significantly higher in the OGF group and the FMT group compared with the CUMS group (*P* < 0.05, *P* < 0.01), and there was no significant difference in the expression of each protein in the ABX and ABX + OGF groups (*P* > 0.05).

### TLR4/NF-κB/NLRP3 pathway identified

#### Transcriptome sequencing analysis results

**FIGURE 5:**
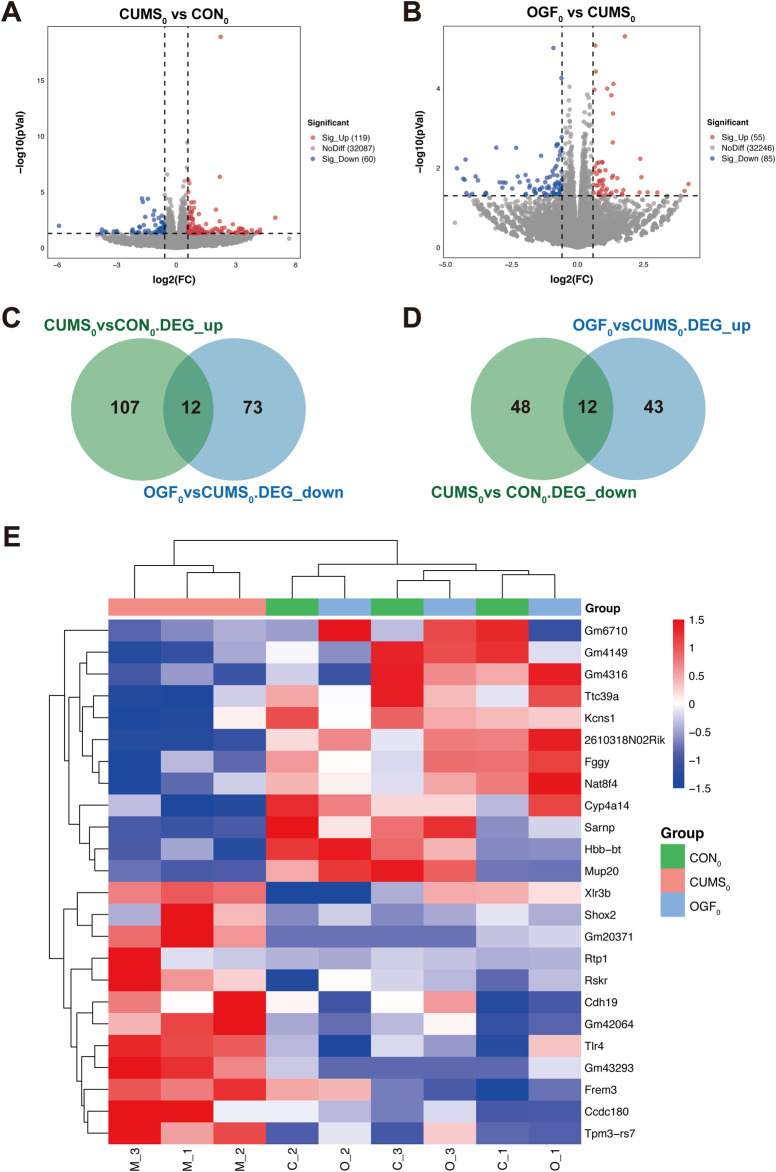
Results of transcriptomic data analysis based on preliminary experiments **(A)** Volcano plot of DEGs in CUMS_0_ vs. CON_0_. **(B)** Volcano plot of CUMS_0_ vs. CON_0_. **(C)** Intersection of upregulated DEGs in CUMS_0_ vs. CON_0_ with downregulated DEGs in OGF_0_ vs. CUMS_0_. **(D)** Intersection of downregulated DEGs in CUMS_0_ vs. CON_0_ with upregulated DEGs in OGF_0_ vs. CUMS_0_. **(E)** Heatmap of 24 differential gene expression clusters in the hippocampus of different groups of mice.

According to the statistical and comparative analysis of the sequencing data after quality control, these data were of high quality, indicating that the sequencing was effective. The results are shown in Supplementary Tables S1, S2. The results of differentially expressed gene analysis are shown in Figures 5A,B. Compared to the CON_0_ group, the CUMS_0_ group had 179 DEGs, with 119 upregulated and 60 downregulated genes. Compared to the CUMS_0_ group, the OGF_0_ group had 140 DEGs, with 55 upregulated and 85 downregulated genes.

As shown in Figures 5C,D, the above two groups of upregulated and downregulated DEGs were intersected to obtain 24 DEGs, A total of 12 genes were upregulated in the CUMS_0_ group and downregulated in the OGF_0_ group, namely, *Shox2*, *Rtp1*, *Ccdc180*, *Rskr*, *Tlr4*, *Frem3*, *Cdh19*, *Tpm3-rs7*, *Xlr3b*, *Gm43293*, *Gm20371*, *Gm42064*. A total of 12 genes were downregulated in the CUMS_0_ group and upregulated in the OGF_0_ group, namely, *Ttc39a*, *Fggy*, *Sarnp*, *Kcns1*, *2610318N02Rik*, *Nat8f4*, *Hbb-bt*, *Mup20*, *Cyp4a14*, *Gm4149*, *Gm6710, Gm4316*.

The expression levels of 24 DEGs were clustered, and the heat map showed that these DEGs were reversed to varying degrees after OGF administration. Moreover, samples from the OGF_0_ group were found to be clustered with samples from the CON_0_ group, while those from the CUMS_0_ group were found to cluster separately (Figure 5E).

#### Depression-related targets and gene chip DEGs

**FIGURE 6:**
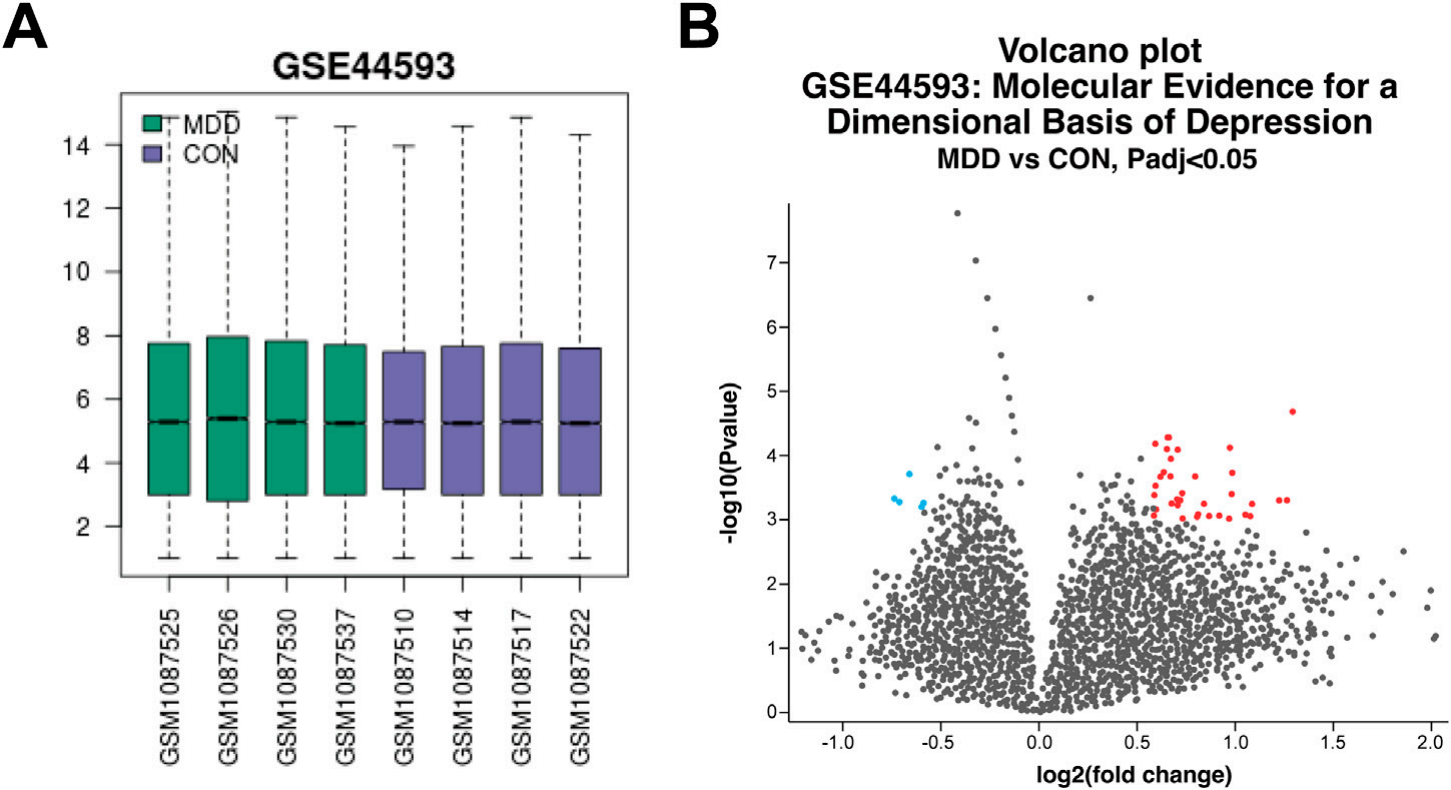
Quality analysis of GeneChip samples and screening of DEGs **(A)** Box plots of each sampled value. **(B)** Volcano plot of DEGs between depressed and control patients.

A total of 2116 depression-related targets were obtained by searching the Genecards, OMIM, and CTD databases. The values of the selected samples from the GSE44593 dataset were represented by a box plot (Figure 6A), which showed that the median and quartiles of the samples were at the same level, indicating that the quality of the gene chips was good. Differential gene screening analysis was then performed to obtain 40 DEGs (Figure 6B).

#### Identify core target genes and associated pathways

**FIGURE 7:**
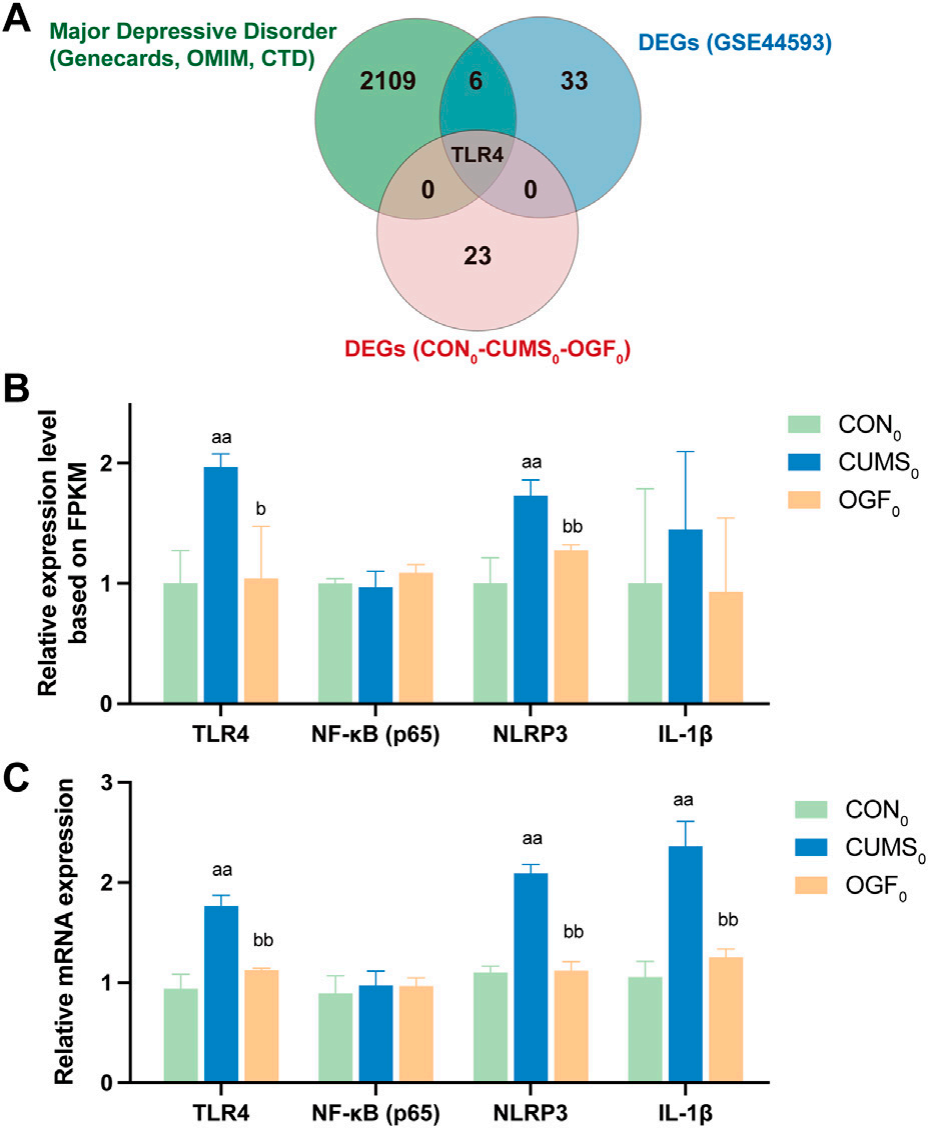
Identification of core target genes and the TLR4/NF-κB/NLRP3 signaling pathway **(A)** Intersection of depression-related targets, DEGs from gene chip and DEGs from transcriptome data. **(B)** Relative gene expression based on transcriptome FPKM (n = 3). **(C)** The relative expression of each gene was determined based on qPCR experiments (n = 6). ^a^ *P* < 0.05, ^aa^ *P* < 0.01 *versus* CON_0_ group; ^b^ *P* < 0.05, ^bb^ *P* < 0.01 *versus* CUMS_0_ group.

In Figure 7A, the DEGs were intersected with the depression-related targets and gene chip DEGs and finally all three were intersected together in the core target, TLR4. As LPS in serum can stimulate the downstream TLR4/NF-κB/NLRP3 signaling pathway, ultimately leading to neuroinflammation (Tong et al., 2020). Therefore, the pathway was validated by transcriptomic data (gene expression FPKM) and qPCR experiments. The results (Figures 7B,C) all indicated that the relative expression levels of *Tlr4*, *Nlrp3*, and *Il-1β* genes in the hippocampus of depressed mice showed reversal trend after OGF administration. Since NF-κB activation depended on phosphorylation of p65 as well as nuclear translocation, and the form of phosphorylation modification belonged to the post-translational modification part of the protein and did not exist at the gene level, the subsequent detection of p-NF-κB expression level at the protein level was required.

### Effects of fecal microbial transplantation on the hippocampal TLR4/NF-κB/NLRP3 pathway in CUMS-induced mice

**FIGURE 8:**
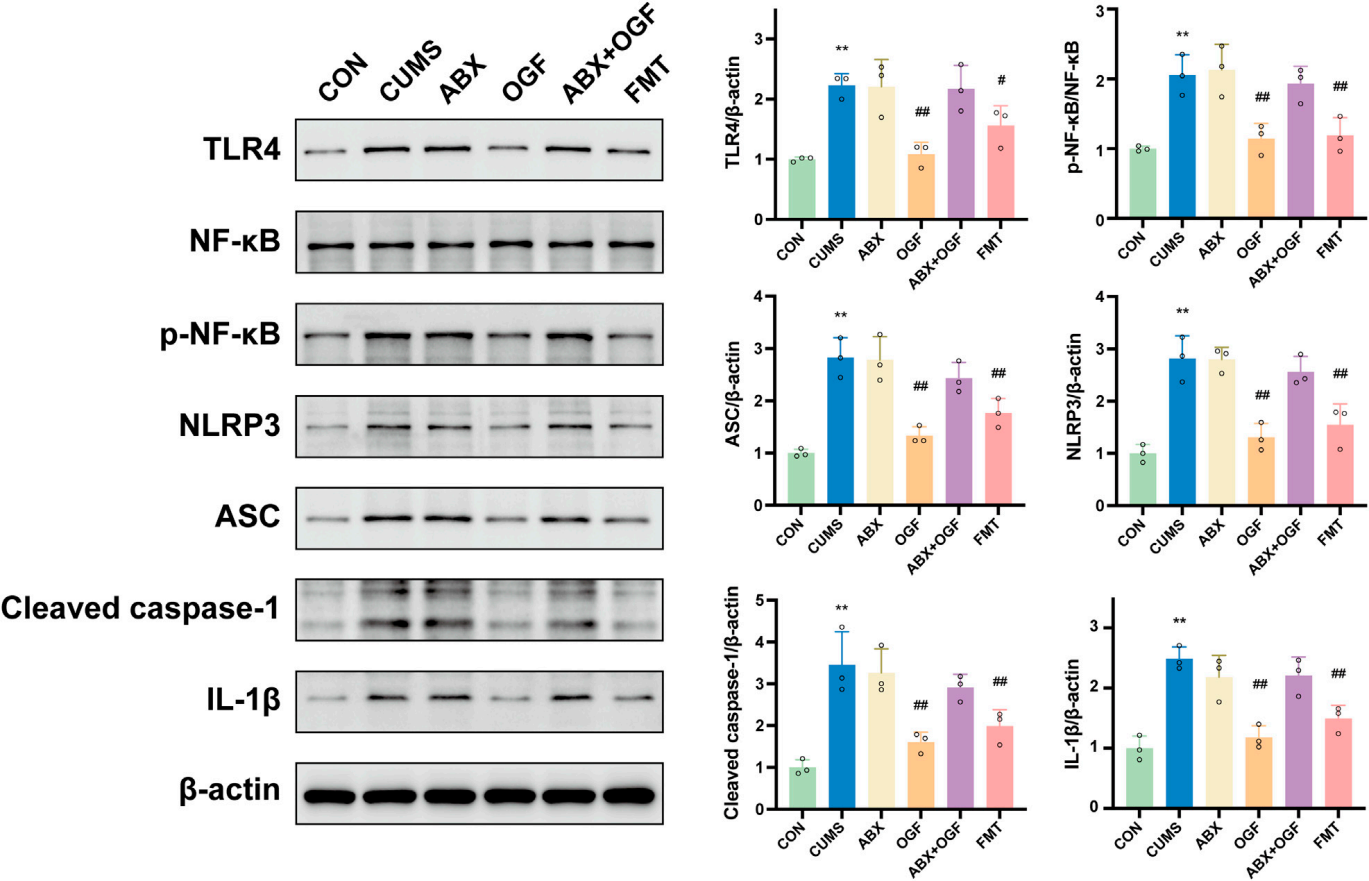
Expression of TLR4/NF-κB/NLRP3 pathway-related proteins in the hippocampus of different groups of mice.

As shown in Figure 8, protein levels of TLR4, p-NF-κB, NLRP3, ASC, and IL-1β were significantly upregulated in the hippocampus of mice in the CUMS group compared with the CON group (*P* < 0.01), and caspase-1 (P 45) was significantly activated as cleaved caspase-1 (P 20, *P* < 0.01). The OGF and FMT groups significantly reversed this (*P* < 0.05 or *P* < 0.01) but there was no significant difference in the expression of pathway-related proteins between the ABX, ABX + OGF, and CUMS groups (*P* > 0.05).

## Discussion

Depression has been identified as a significant global mental health concern, impacting both physical and mental wellbeing. The team has previously demonstrated that the study of OGF can ameliorate depressive-like behavior and improve the intestinal microbiota dysbiosis in CUMS mice (Han et al., 2024). The present study was thus designed to investigate further the gut-brain axis-mediated antidepressant effect of OGF and its mechanism of action.

According to the results of behavioral tests, both direct OGF treatment and FMT treatment significantly improved depressive-like behavior in mice; the same trend was observed in the SPT, FST, TST, NSFT, OFT, and EPM, providing strong evidence for their antidepressant effects. However, the intervention of antibiotic treatment (ABX group and ABX + OGF group) did not show any improvement; through comparative analysis of the various test items, it was found that their effects on depressive-like behavior were unstable; the addition of antibiotics also led to a slight decrease in mouse body weight, and these adverse effects were effectively improved after fecal microbiota transplantation.

The CUMS has been demonstrated to effectively induce depression-like behavioral and physiological changes, thus serving as a widely accepted model for evaluating antidepressant medications (Willner, 2017; Zhou et al., 2022). The etiology of depression is thought to be multifactorial, involving dysregulation of neuroplasticity like hippocampal atrophy and neuronal reduction, monoamine neurotransmitters, neurotrophic factors, inflammation, and dysregulation of gut microbiota (Monroe and Harkness, 2022). A salient finding is the observation of hippocampal atrophy and pyramidal cell reduction in the brains of depressed patients (Roddy et al., 2019). Specifically, the volume of the left CA1 region of the hippocampus has been identified as a potential marker for depression, with the CA2/3/4 regions implicated in the initial onset of the condition and exhibiting development alongside the process. Neurotransmitters, defined as substances that facilitate the transmission of information between nerve cells, are crucial for maintaining optimal mood, cognitive function, and behavior (Monroe and Harkness, 2022). Research findings have indicated that the synaptic gap-closing neurotransmitters 5-HT and DA are significantly reduced in depressed patients compared to healthy individuals. BDNF, a pivotal member of the neurotrophic factor family, has been demonstrated to influence neuronal growth, development, differentiation, and survival, thereby impacting brain function (Kim et al., 2007). The investigation of the correlation between BDNF levels and the severity of depression in patients with major depressive disorder reveals a negative association between low BDNF levels and the intensity of depression symptoms. The concept of neuroinflammation as a protective response in the brain is postulated; nevertheless, social and psychological stress have been shown to cause the inflammatory system to become overactive, thereby increasing the risk of developing depression (Beurel et al., 2020). Clinical studies have identified elevated levels of pro-inflammatory cytokines (IL-1β, IL-6, TNF-α) in brain tissue and serum of depressed patients, and depressive symptoms in healthy individuals treated with inflammatory cytokines or related inducers, which can be blocked by anti-inflammatory interventions. In the present study, both OGF and FMT of OGF-treated mice exhibited significant antidepressant effects, including improvements in body weight and depressive-like behaviors, attenuation of hippocampal tissue damage, restoration of hippocampal BDNF and neurotransmitter levels of 5-HT and DA, and reduction of serum IL-1β, IL-6, and TNF-α levels in CUMS-induced mice.

The microbial gut-brain axis, which refers to the influence of gut microbiota on the development of depression, may be mediated by the modulation of the immune-inflammatory response, neurotransmitters, and other metabolites involved in the bidirectional regulation of the gut and central nervous system (Socała et al., 2021). Intestinal inflammation, triggered by dysbiosis, has been shown to induce the release of pro-inflammatory cytokines, which have been demonstrated to compromise the integrity of the intestinal and blood-brain barriers, ultimately resulting in neuroinflammation. In this study, both OGF and FMT demonstrated significant efficacy in ameliorating intestinal barrier damage. This included the attenuation of pathological damage and inflammatory infiltration of colonic tissues, a decrease in serum DAO and LPS concentrations, and an increase in the expression levels of colonic tight junction proteins ZO-1, Occludin, and Claudin-1.

FMT is a therapeutic approach in which fecal bacteria from a donor are transplanted into the gastrointestinal tract of a recipient to reconstitute the recipient’s gut microbiota. This approach has gained significant traction in the clinical management of psychiatric disorders (Sonali et al., 2022). At the same time, transplanting the flora of a drug-taking donor to a recipient to indirectly ameliorate the disease is a well-recognized method of determining whether a drug acts through the intestinal flora (Belvoncikova et al., 2022). The eradication of the recipient’s native microbiota through the administration of antibiotics has emerged as a prevalent model for “pseudo-sterile” recipients. This approach ensures pre-transplant intestinal cleansing and enhances the colonization ability of the donor microorganisms. In the previous study, the results of 16S rRNA sequencing proved that OGF improved the intestinal flora disorder in depressed mice. At the genus level, OGF significantly increased the abundance of *Muribaculaceae* and *Eubacterium_ventriosum_group* and significantly decreases the abundance of *Lachnospiraceae_NK4A136_group*, *Ruminococcus_torques_group*, and *Acetitomaculum*. These microbiomes have been shown to be closely associated with the onset and progression of depression in animal experiments and clinical studies. In this study, fecal microbial transplantation from OGF-treated mice exhibited significant therapeutic effects in assessing depression-like behavior, hippocampal tissue damage and related index levels, and colonic barrier function in all groups of mice. It is noteworthy that this effect was observed to be second only to direct OGF treatment. However, when depressed mice were pre-treated with antibiotics to eliminate intestinal microorganisms, the subsequent OGF treatment (ABX + OGF) did not significantly improve the aforementioned situation. Consequently, the study concluded that OGF’s antidepressant effects are facilitated by the intestinal microbiota.

**FIGURE 9:**
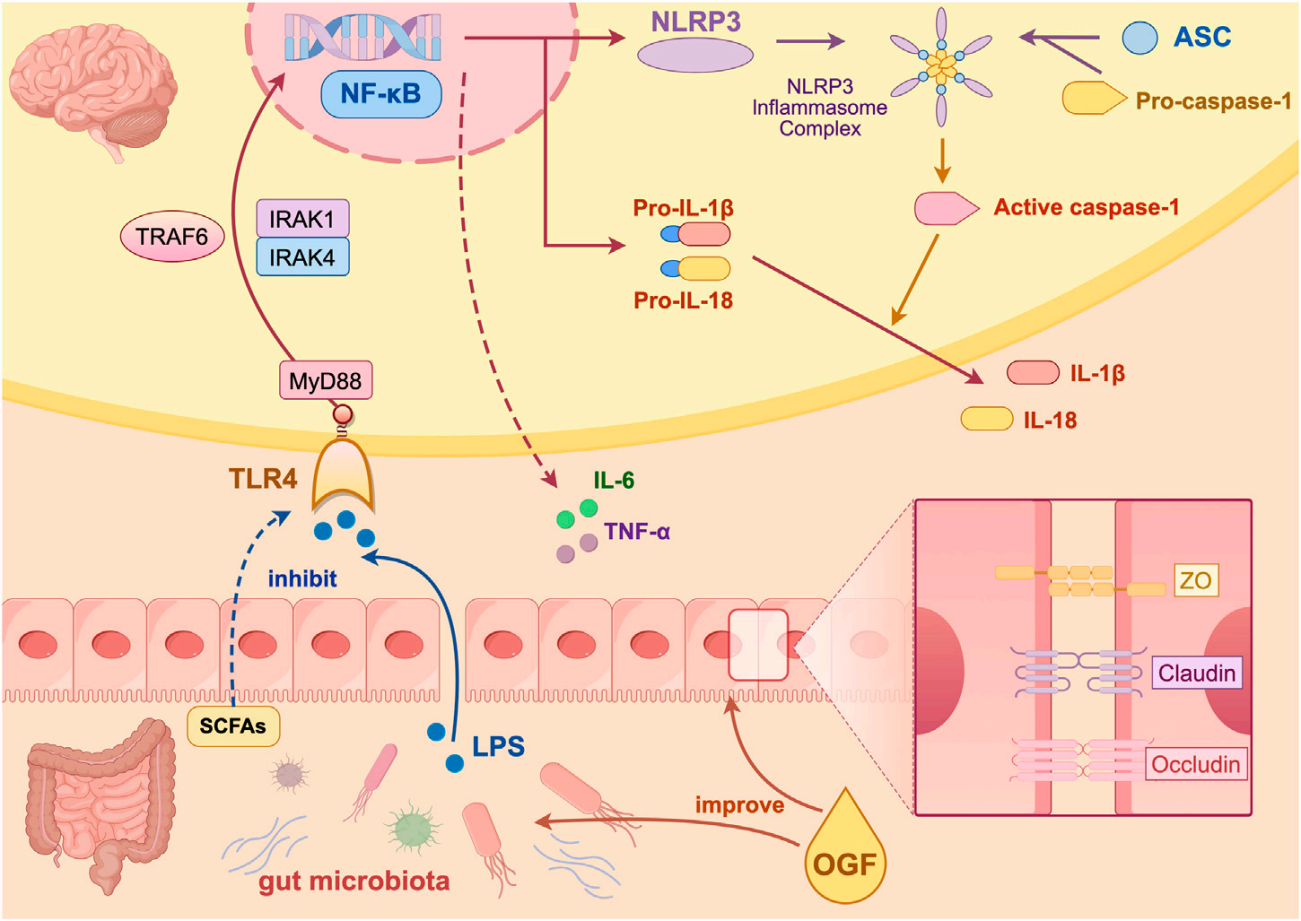
Mechanism diagram of the antidepressant effect of OGF created on Figdraw.

In order to explore the signaling pathways regulated by OGF in improving depression-like behavior, the group screened the core target TLR4 by combining the sequencing data from the previous animal experiments with human clinical data, and jointly verified the TLR4/NF-κB/NLRP3 pathway by combining the gene level and protein level. Increased intestinal permeability allows for enhanced penetration of the bloodstream by LPS, a component of the cell membrane of Gram-negative bacteria. This heightened permeability facilitates the entry of LPS into the brain, inducing neuroinflammation (Swanson et al., 2019; Tong et al., 2020). This process is primarily driven by the activation of the NLRP3 inflammasome via the TLR4/NF-κB pathway. The sequence of events begins with LPS binding to TLR4, followed by its recognition and delivery. This process culminates in NF-κB nuclear translocation and the subsequent production of inflammatory factors. The assembly and activation of the NLRP3 inflammasome (comprising NLRP3, ASC, and caspase-1) is initiated by various exogenous and endogenous stimuli, resulting in the secretion of IL-1β and IL-18, thus inducing inflammation. In the present study, FMT from patients treated with OGF was found to modulate the CUMS-induced activation of the TLR4/NF-κB/NLRP3 pathway. Conversely, pretreatment with OGF did not demonstrate any significant modulation of the pathway when administered with antibiotics. This finding suggested that the absence of the microbiota could not alleviate hippocampal inflammation, and the modulation of TLR4/NF-κB/NLRP3 pathway by OGF might be associated with intestinal microbiota (Figure 9).

By comparison, it can be found that previous studies focused more on the antidepressant efficacy of different doses of OGF and its effects on the gut microbiota. This study focused more on the connection between antidepressant effect and the microbiota to explore whether OGF exerts its antidepressant effects through the gut microbiota, including restoring damaged colon barriers and inhibiting neuroinflammation.

## Conclusion

In summary, OGF could exert its antidepressant effects by regulating the gut microbiota and improving intestinal barrier function, thereby mediating the hippocampal TLR4/NF-κB/NLRP3 pathway to alleviate inflammatory responses in CUMS-induced mice. This study provides substantial evidence that OGF exerted an antidepressant effect by the gut-brain axis, thereby establishing a scientific foundation for its further development and utilization. Based on this research, it is expected that OGF will be developed in the form of microcapsules or probiotics in the future and clinical research will be conducted jointly with hospitals.

## Data Availability

The datasets presented in this study can be found in online repositories. The names of the repository/repositories and accession number(s) can be found below: GEO dataset and GSE44593. The raw FPKM data, including the expression levels of all genes in the hippocampus of each mouse in each group, is submitted for public access in Supplementary Data Sheet 2.

## Abbreviations

ANOVA, One-way analysis of variance; BDNF, Brain-derived neurotrophic factor; CUMS, Chronic unpredictable mild stress; DAO, Diamine oxidase; DEGs, Differentially expressed genes; DA, Dopamine; EPM, Elevated plus-maze test; ELISA, Enzyme-linked immunosorbent assays; FMT, Fecal microbiota transplantation; FST, Forced swimming test; HE, Hematoxylin-eosin; IL-1β, Interleukin-1β; IL-6, Interleukin-6; MGB, Microbiota-gut-brain; NSFT, Novelty-suppressed feeding test; OGF, Gardeniae Fructus oil; OFT, Open field test; SPT, Sucrose preference test; TST, Tail suspension test; TNF-α, Tumor necrosis factor-α; LPS, Lipopolysaccharide; 5-HT, 5-hydroxytryptamine.
