# Supplementary material for: Modulation of gut microbiota by Gardeniae Fructus oil exerts TLR4/NF-κB/NLRP3 pathway-mediated antidepressant effects based on transcriptomics and fecal transplantation
Source: Front Pharmacol. 2025 Aug 1;16:1635897. doi: 10.3389/fphar.2025.1635897 (PMC12354470; doi:10.3389/fphar.2025.1635897)
Supplement: Supplementary file 1 [file DataSheet1.docx]

***Supplementary material***


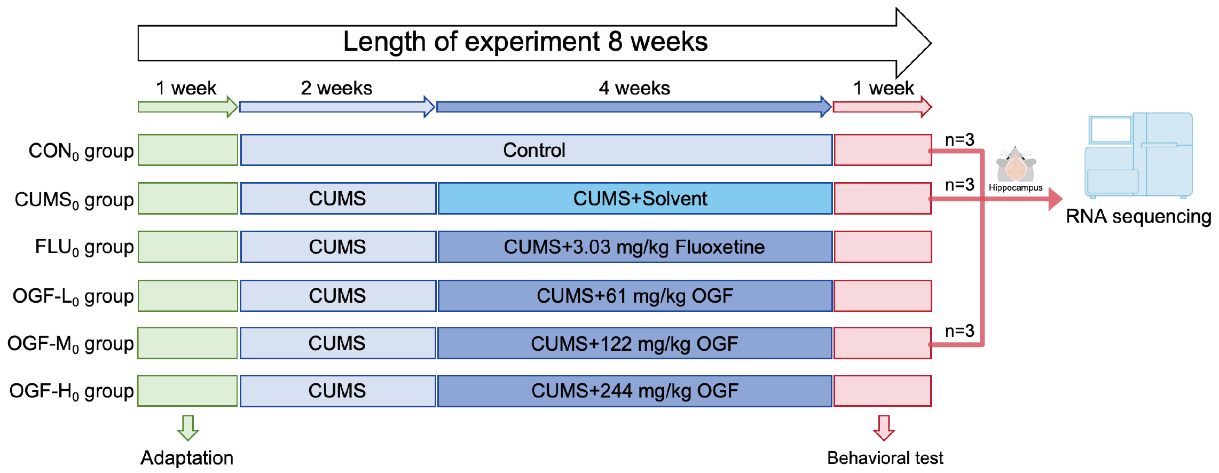


**Figure S1** Flowchart of preliminary animal experiments on OGF.

**Table S1** Statistical Table of Sequencing Data

| Sample name | Raw sequencing data | |  | Processed sequencing data | | | | | |
| --- | --- | --- | --- | --- | --- | --- | --- | --- | --- |
|  | Reads count | Total base count |  | Reads count | Total base count | Error rate（%） | Q20（%） | Q30（%） | GC content (%) |
| C_1 | 45968668 | 6.94G |  | 45564800 | 6.86G | 0.02 | 97.98 | 96.10 | 48.34 |
| C_2 | 45686766 | 6.90G |  | 45356802 | 6.83G | 0.02 | 98.20 | 96.50 | 48.26 |
| C_3 | 37960602 | 5.73G |  | 37588336 | 5.66G | 0.02 | 97.95 | 95.98 | 48.18 |
| M_1 | 41718108 | 6.30G |  | 41380848 | 6.23G | 0.02 | 98.06 | 96.24 | 47.94 |
| M_2 | 46352000 | 7.00G |  | 45992380 | 6.92G | 0.02 | 98.21 | 96.48 | 47.80 |
| M_3 | 38530824 | 5.82G |  | 38209446 | 5.75G | 0.02 | 98.09 | 96.29 | 48.08 |
| O_1 | 37963840 | 5.73G |  | 37679558 | 5.67G | 0.02 | 98.21 | 96.49 | 48.08 |
| O_2 | 43170776 | 6.52G |  | 42837736 | 6.44G | 0.02 | 98.06 | 96.25 | 48.54 |
| O_3 | 42873172 | 6.47G |  | 42491852 | 6.40G | 0.02 | 98.06 | 96.21 | 47.99 |

**Table S2** Statistical Table of Gene Matching Rates

| Sample name | Total read count | Total alignment rate (%) | Multiple alignment rate (%) | Unique alignment rate (%) |
| --- | --- | --- | --- | --- |
| C_1 | 45564800 | 97.40 | 4.89 | 92.52 |
| C_2 | 45356802 | 97.54 | 5.11 | 92.42 |
| C_3 | 37588336 | 97.07 | 4.85 | 92.21 |
| M_1 | 41380848 | 97.28 | 5.10 | 92.19 |
| M_2 | 45992380 | 97.31 | 5.09 | 92.23 |
| M_3 | 38209446 | 97.26 | 4.83 | 92.43 |
| O_1 | 37679558 | 97.28 | 4.90 | 92.39 |
| O_2 | 42837736 | 97.33 | 4.92 | 92.41 |
| O_3 | 42491852 | 97.20 | 4.95 | 92.26 |
